# Th-17 regulatory cytokines inhibit corticosteroid induced airway structural cells apoptosis

**DOI:** 10.1186/s12931-015-0307-2

**Published:** 2016-01-16

**Authors:** Rabih Halwani, Asma Sultana, Roua Al-Kufaidy, Amer Jamhawi, Alejandro Vazquez-Tello, Saleh Al-Muhsen

**Affiliations:** Prince Naif Center for Immunology Research and Asthma Research Chair, Department of Pediatrics, College of Medicine, King Saud University, P. O. Box 2925, Postal Code 11461 Riyadh, Saudi Arabia; Prince Naif Health Research Center, King Saud University, Riyadh, Saudi Arabia

## Abstract

**Background:**

Although corticosteroid is a powerful anti-inflammatory drug that is used widely to control asthma, still severe asthmatics can develop steroid resistance. Airway fibroblasts are quite resistant to steroids during Idiopathic pulmonary fibrosis (IPF) and fibrosis in asthmatic lungs is not always controlled. Th-17 regulatory cytokine which are elevated in lung tissues of asthmatics were shown to enhance the survival of various types of cells. STAT factors are central to this anti-apoptotic function. However, it is not yet clear whether these cytokines contribute to steroid hypo-responsiveness in asthma. Therefore, in this study, we investigated the ability of Th-17 regulatory cytokines, specifically IL-21, IL22 and IL23, to protect structural airway cells against dexamethasone-induced apoptosis.

**Methods:**

Primary human fibroblasts, ASM cells, and lung endothelial cells line were treated with IL-21, IL-22, and IL-23 cytokines before incubation with dexamethasone and the level of apoptosis was determined by measuring cellular Annexin-V using Flow cytometry.

**Results:**

Our data indicated that treatment with Th-17 regulatory cytokines was effective in inhibiting induced apoptosis for both fibroblasts and endothelial cells but not ASM cells. STAT3 phosphorylation levels were also upregulated in fibroblasts and endothelial upon treatment with these cytokines. Interestingly, inhibiting STAT3 phosphorylation abrogated IL-21, IL-22, and IL-23 anti-apoptotic effect on fibroblasts and endothelial cells.

**Conclusions:**

This data suggest that Th-17 regulatory cytokines may play a critical role in regulating the survival of fibroblasts during asthma, IPF as well as other chronic lung inflammatory diseases leading to enhanced fibrosis. Accordingly, findings of this paper may pave the way for more extensive research on the role of these regulatory cytokines in fibrosis development in various chronic inflammatory diseases.

## Background

Naturally occurring glucocorticoids (e.g., cortisol) and their synthetic derivatives (e.g., dexamethasone) are a class of powerful anti-inflammatory steroid molecules in use since several decades to keep inflammatory processes under control [1]. Despite the risks of side effects, glucocorticoids remain up-to-date the most widely prescribed and effective agents for the treatment of chronic inflammation of the airways such as asthma and COPD [2, 3]. In the airway tissues of asthmatics, improvement of the inflammatory state results, at least in part, from the glucocorticoid-induced apoptosis of infiltrated pro-inflammatory cells of lymphoid (e.g., T lymphocytes, NK cells) and myeloid (e.g., eosinophils, macrophages) lineages.

A common characteristic of asthmatic patients is airway tissue remodelling, that seemingly result from attempted healing processes by injured tissue after chronic exposure to environmental irritants [4, 5]. With a dysregulated tissue homeostasis, structural cells of the airways, namely, smooth muscle cells, fibroblasts and endothelial cells, release many mediators, including chemokines that attract circulating pro-inflammatory leukocytes, such as granulocytes. Even though glucocorticoid treatment helps to control airway inflammation, airway remodelling could be enhanced by high-doses or chronic (long-term) exposure to these drugs [6, 7]. In fact, airway epithelium damage in asthmatics is promoted by glucocorticoid therapy, by inducing epithelial cell apoptosis and suppressing its proliferation, thus concomitantly hindering epithelium repair and likely contributing to airway remodelling [8–10]. Similar pro-apoptotic effect was observed on fibroblasts exposed to increased concentrations of glucocorticoid [11, 12].

Several cytokines has been reported to play a role in steroid resistance [13, 14]. For instance, Th-17-derived IL-17 cytokines can hamper both anti-inflammatory and immunosuppressant actions of dexamethasone on peripheral lymphocytes, in part via a mechanism that upregulates glucocorticoid-receptor beta (GR-β) [15]. Epithelial cells can also be protected against dexamethasone-induced apoptosis by Th-2 cytokines IL-9 and IL-13, via activation of signal transducer and activator of transcription factors (STAT1, STAT3 and STAT5) and upregulation of the anti-apoptotic Bcl-2 gene [16]. Airway fibroblasts are quite sensitive to steroids, so much that when incubated in vitro with dexamethasone, die within a few hours [12]; however, fibrosis in asthmatic patients is not always successfully controlled, suggesting that alternative protective anti-apoptotic mechanisms may be involved [4, 17].

In the lung tissues of severe asthmatics, a preferential infiltration of Th-17 cells and elevated levels of various cytokines (e.g., IL-17, IL-21, IL-22, IL-23) is usually observed [18–22]. It is not yet clear whether Th-17-derived cytokines contribute to steroid hypo-responsiveness, such as protection against apoptosis. Lajoie et al. have recently demonstrated that IL-21-positive cells are increased in the bronchial mucosa of asthmatics compared with non-asthmatics [19]. They have shown that IL-21 plays a critical role in enhancing airway inflammation and hyper-responsiveness (AHR) in mice asthma model. IL-21R-deficiency reduced HDM-driven AHR and resulted in significant suppression in protein levels of the Th2 cytokines IL-4, and IL-13. Similarly, Jin et al. reported that IL-21 is a critical regulator of the processes that lead to skin sensitization and allergic inflammation [23]. IL-22, a member of the IL-10 family cytokines that is mainly secreted by Th17/Th22 cells, plays critical roles in innate and adaptive immunity.

IL-22 protects the liver against damage and favours its regeneration by inducing the expression of mitogenic and anti-apoptotic proteins in hepatocytes and liver stem cells [24]. Recently, IL-22 was found to have immune modulatory effects on pulmonary allergic inflammation [25, 26] although this role is still controversial. Serum levels of IL-22 has been found to be elevated in the blood of asthmatic patients and lung tissues of asthma mouse model [20] and to correlate with disease severity [21, 27, 28]. Other reports indicated that IL-22 may have immune modulatory effects on the development of allergen-induced pulmonary inflammation [20, 29–31]. IL-23 signalling plays an important role in the pathogenesis of severe and steroid-resistant asthma through the modulation of Th2 cell differentiation. In fact, transgenic overexpression of IL-23R in allergen-induced asthmatic mice promoted increased airway infiltration of eosinophils and Th2 cytokine production [32]. Moreover, an inverse correlation between IL-23 and FEV1 has been demonstrated in asthmatic children [33, 34]. Interestingly, increased expression of IL-23 in allergic airway inflammation has been confirmed and ova induced asthmatic inflammation of mice could be reversed by inhibition of the IL-23 signalling pathway [22].

Th-17-derived IL-17A, IL-17F and IL-22 cytokines can also enhance survival of primary airway smooth muscle cells in culture, by reducing spontaneous apoptosis, although this protective effect was not tested in the presence of steroids [35]. IL-21 and IL-23.

IL-21 has a pro-apoptotic effect on B-cells and follicular lymphoma [36, 37], but promotes cell growth and proliferation in T cells [38, 39], especially during T-cell leukaemias, myeloma, [37, 40, 41], and lymphomas [42]. Similarly, although IL-23 induces apoptosis in several leukocytes, [43], it promoted survival of memory T cells via the activation of Stat pathways [44, 45]. However, no information is available of a potential pro- or anti-apoptotic effect of both IL-21 and 23 on primary airway structural cells.

IL-21 was shown to act as a long-term growth and survival factor in MM cell lines where only STAT3 but not STAT1 was activated [40]. The main mechanism mediating IL-22 immune responses involves the activation of anti-apoptotic or proliferative genes. IL-22 mainly activates Jak-STAT-particularly STAT3 signalling pathways, in addition to MAPK-Akt, and Bcl-2, which are critical for IL-22 functions [46]. In addition, several reports indicated that excessive IL-22 can lead to tumor growth, inhibition of apoptosis, and promotion of metastasis [47, 48]. This effect of IL-22 is mostly due to its ability to activate STAT3. Similarly, IL-23 regulated the growth of human lung cancer cells through its effects on STAT3 expression and phosphorylation in a concentration-dependent manner [49].

IL-17A and IL-17F have been indicated previously, in several studies, to enhance lung fibroblast survival [35, 50, 51]. We have, hence, focused on other Th-17 regulatory cytokines as many are also upregulated in severe asthma but their role in maintaining lung structural cells is not properly investigated. Therefore, in this study, we investigated the possible contribution of IL-21, IL-22 and IL-23, to protection of structural airway cells against dexamethasone-induced apoptosis and whether the anti-apoptotic mechanism involves STAT transcription factor activation.

## Methods

### Primary cell culture

Primary airway smooth muscle cells (ASMCs, Discovery Biomed Inc.) were grown in 1:1 combination of complete Advanced MEM and Vasculife media in 5 % CO_2_ incubator at 37 °C. Human primary lung fibroblasts obtained by bronchoscopy of healthy subjects were grown in DMEM medium supplemented with 10 % FBS, 10 U/ml penicillin, 10 μg/ml streptomycin, and 62.5 ng/ml fungizone (Invitrogen) and cultured at 37 °C, under an atmosphere containing 5 % CO_2_. HMVEC-L endothelial cells were cultured in RPMI-1640 medium supplemented with 10 % FBS and 10 U/ml penicillin, 10 μg/ml streptomycin.

### Cell viability assessment assay

To determine if Th-17 regulatory cytokines (IL-21 and IL-23) as well as IL-22 exert a protective effect against dexamethasone-induced apoptosis, ASMCs, endothelial cells and fibroblasts were cultured in their respective media in 6-well plates at a seeding density of 60,000 cells/well. To determine Dexamethasone concentration to be used, a range of 0.1, 0.5, 1, 2, 5 and 10 μM was tested for apoptotic effect. Cellular apoptosis was observed at concentration as low as 0.1 μM and no increase in apoptosis was noticed above 5 μM. A range of cytokine concentrations were tested for anti-apoptotic effect and the concentration with max effect on dexamethasone (5 μM) treated cells (above which no decrease in apoptosis was observed) was chosen. Cells were stimulated or not with cytokines IL-21, IL-22, IL-23, IL-21+IL-22, IL-21+IL-23, IL-22+IL-23, IL-21+IL-22+IL23, and IL-6 (50 ng/mL each) for 1 h. Dexamethasone (5 μM) was added to cytokine treated cells and incubation continued for 24 h. Early apoptotic cells were stained for 15 min in the dark at room temperature with Annexin V-APC (1 μg/mL, 550474, BD Biosciences) and propidium iodide (1 μg/mL, PI, P3566, Invitrogen) and immediately analysed using the BD LSRII flow cytometer (BD Biosciences). Early apoptotic cells were considered as those stained with Annexin V-APC only, whereas those stained with both Annexin V-APC and PI were considered as late apoptotic-early necrotic; those stained with PI only were considered as necrotic.

### Phosflow assay for STAT3 phosphorylation

ASMCs, endothelial cells and fibroblasts were cultured to confluence and starved in medium containing 0.5 % FBS for 18 h. Cells were distributed at a cell density of 10^6^ cells/mL and treated or not with dexamethasone (5 μM) for 1 h, then stimulated with cytokines as described above for 15 min. Cells were then stained using Phosflow technique with PE labeled-anti-p-STAT3 antibody (2 μg/10^6^ cells) and analysed using the BD LSRII cytometer (BD Biosciences).

To confirm the role of tyrosine-phosphorylated STAT3 (p-STAT3) as key element in cytokine induced cell protection to dexamethasone-induced apoptosis, we used a selective inhibitor AS601245, which was reported to cause a dose-dependent decrease in p-STAT3 [52]. Primary lung fibroblasts were starved for 18 h in DMEM containing 0.5 % FBS and then stimulated or not for one hour with IL-21+IL-22+IL-23 cytokines at 50 ng/ml each in the presence or absence of the inhibitor (AS601245, 2.5 μM). The cells were then treated with Dexamethasone (5 μM/well) and incubated for further 24 h. After incubation, cells were processed for Annexin V-Propidium Iodide (PI) and flow cytometry analyses as described above.

### Western analysis

Cytokine-induced STAT3 phosphorylation status was also analysed by Western blotting. To this end, 0.3 × 10^6^ endothelial cells and fibroblasts were cultured, starved and stimulated or not for 15 min with cytokines IL-21, IL-22, IL-23, IL-21+IL-22+IL23, and IL-6 as described above. After stimulation, cells were harvested and treated with RIPA lysis buffer (20–188, Upstate cell signalling solutions, CA, USA) containing protease inhibitors. The supernatants were collected, protein concentrations were determined and samples (20 μg/well) were resolved on a 10 % SDS-PAGE. Membranes were then incubated overnight at 4 °C with Tyrosine-phosphorylated and non-phophorylated STAT3 Antibodies (R&D systems) and developed with secondary antibody for 1 h at room temperature. For Stat3 nuclear translocation, fibroblasts were treated or not with dexamethasone (5 μM) for 1 h, then stimulated with cytokines as described above. To fractionate cells into nuclear and cytoplasmic fractions, they were resuspended in hypotonic buffer (Tris pH = 7.5) supplemented with 0.1 % protease inhibitor cocktail (Sigma Aldrich). After swelling in ice for 20 min, plasma membranes were disrupted by repeated pipetting. Samples were centrifuged at 3000 rpm for 10 min at 4 °C to collect cytoplasmic fractions (supernatants). The pellets were washed and resuspended in RIPA buffer. The nuclear fraction (supernatant) was recovered by micro-centrifugation at the highest speed for 10 min at 4 °C. Equal protein concentrations of cytoplasmic and nuclear fractions were resolved on SDS page and analysed using non-phophorylated STAT3 antibodies (R&D systems). In addition, anti-Lamin B (nuclear marker) and anti-β-actin antibodies (R&D systems) were used to confirm proper cellular fractionation. Proteins were visualized using the C-Digit® Blot scanner and captured using the Image Studio™ software (Li-Cor Biosciences).

### Statistical analysis

Otherwise specified, data are presented as mean ± SE. Dexamethasone-induced apoptosis was expressed as percentage of treated cells. Statistical significance was evaluated using ANOVA followed by Bonferroni-Dunn post hoc test. Two-way ANOVA was done to determine if significant differences existed in the means between different groups. Values of *p* <0.05 were considered statistically significant.

## Results

### IL-21, IL-22 and IL-23 cytokines protect endothelial cells and fibroblasts from dexamethasone induced apoptosis

**Fig. 1:**
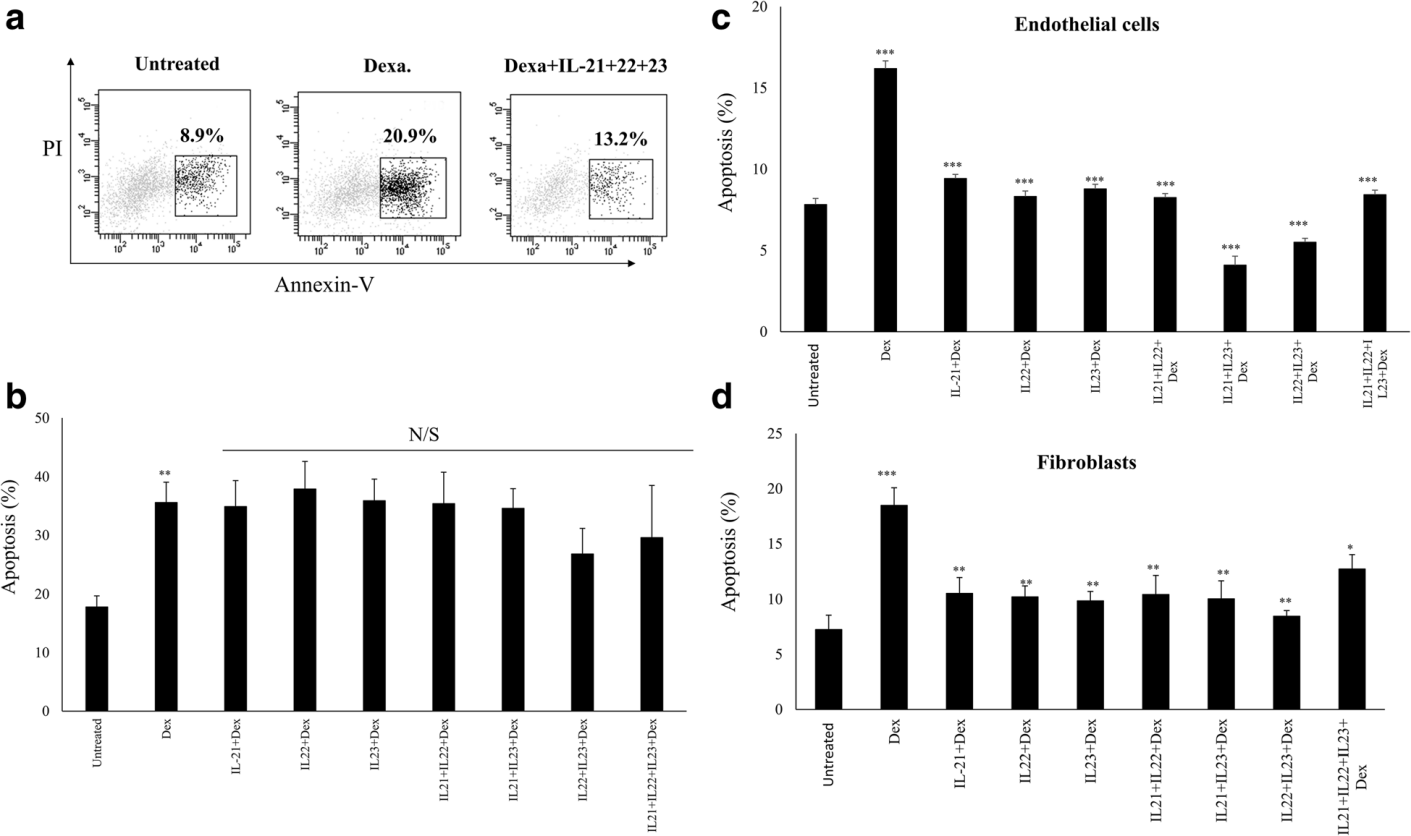
Th-17 regulatory cytokine-induce anti-apoptotic effect on ASMCs, endothelial cells and fibroblasts to dexamethasone. Primary human lung ASMC, fibroblasts, and HMVEC-L endothelial cells were stimulated with individual (IL-21, IL-22, IL-23) and combinations (IL-21+IL-22, IL-22+IL-23, IL-21+IL-23, IL-21+IL-22+IL-23) of cytokines for 1 h and subsequently exposed to dexamethasone (5 μM) for 24 h. The percentage of apoptotic cells were quantified by flow cytometry using Annexin V-PE and PI. Apoptotic cells were categorised as being Annexin V+ve but PI-ve. **a** Representative FACS data showing Th-17 cytokine inhibition of dexamethasone induced apoptosis of fibroblasts. Percentage of apoptosis following treatment with cytokines is shown for (**b**) ASM cells (**c**) Endothelial cells and (**d**) Fibroblasts. (*n* = 8 for each cell type). Apoptosis of cells treated only with dexamethasone were compared to non-treated cells. Apoptosis of cells treated with Dexamethasone and cytokines were compared to cells treated only with Dexamethasone. Data is expressed as means ± SE. * *p* ≤0.05; **p ≤0.01; ***p ≤0.001

The frequency of Th-17 cells and the levels of their regulatory cytokines (IL-21, IL-23) as well as IL-22 were shown to be upregulated in inflamed lung tissues [32, 53, 54]. We, therefore, hypothesized that Th-17 regulatory cytokines, known to promote survival of various cells, could regulate the survival of airway structural cells during chronic inflammatory diseases such as asthma. To that end, we analyzed in vitro, the ability of IL-21, IL-22, and IL-23 cytokines to protect airway structural cells from dexamethasone-induced apoptosis. Human primary lung fibroblasts, ASM cells, as well as HMVEC-L cells were stimulated with the above mentioned cytokines alone or in combinations for 1 h then treated with dexamethasone for 24 h and early apoptotic cells were quantified using FACS analysis. Figure 1a shows a representative data for fibroblasts treated with dexamethasone in the presence or absence of Th-17 cytokines. As expected, dexamethasone treatment induced a substantial increase in apoptosis of cultured ASMCs (35.6 %, Fig. 1b), endothelial cells (16.1 %, Fig. 1c) and fibroblasts (18.5 %, Fig. 1d). However, prior stimulation with IL-21, IL-22 and IL-23 cytokines either alone or in combinations significantly protected the cells from dexamethasone induced apoptosis (Fig. 1; endothelial cells (IL-21: 9.4 %, IL-22: 8.2 %, IL-23:8.78 %, IL21+IL-22: 8.25 %, IL-21+IL-23: 4.1 %, IL-22+IL-23: 5.5 %, IL-21+IL22+IL-23: 8.4 %; all *p* <0.0001); fibroblasts (IL-21: 10.5 % [*p* = 0.010], IL-22: 10.2 % [*p* = 0.006], IL-23:9.9 % [*p* = 0.005], IL21+IL-22: 10.4 % [*p* = 0.01], IL-21+IL-23: 10.0 % [*p* = 0.008], IL-22+IL-23: 8.5 % [*p* = 0.001], IL-21+IL22+IL-23: 12.7 % [*p* = 0.051]). Interestingly, although a decrease in ASM apoptosis was observed following stimulation with IL-22+IL-23 (35.6 % vs 26.8 %) and IL-21+IL22+IL-23 (35.6 % vs 29.6 %) combinations compared to non-stimulated cells, this decrease did not reach significance. The substantial reduction of dexamethasone induced apoptosis of fibroblasts and endothelial cells upon stimulation with IL-21, IL-22 and IL-23 cytokines indicate that these Th-17 cytokines may exert an anti-apoptotic effect on airway structural cells; such an effect could be related to the observed limited anti-inflammatory effect of corticosteroid, or steroid hypo-responsiveness, in some severe asthma patients with predominant Th-17 cytokine profile.

### IL-21, IL-23 and IL-22 cytokines mediate anti-apoptotic cell protection via STAT3 phosphorylation

**Fig. 2:**
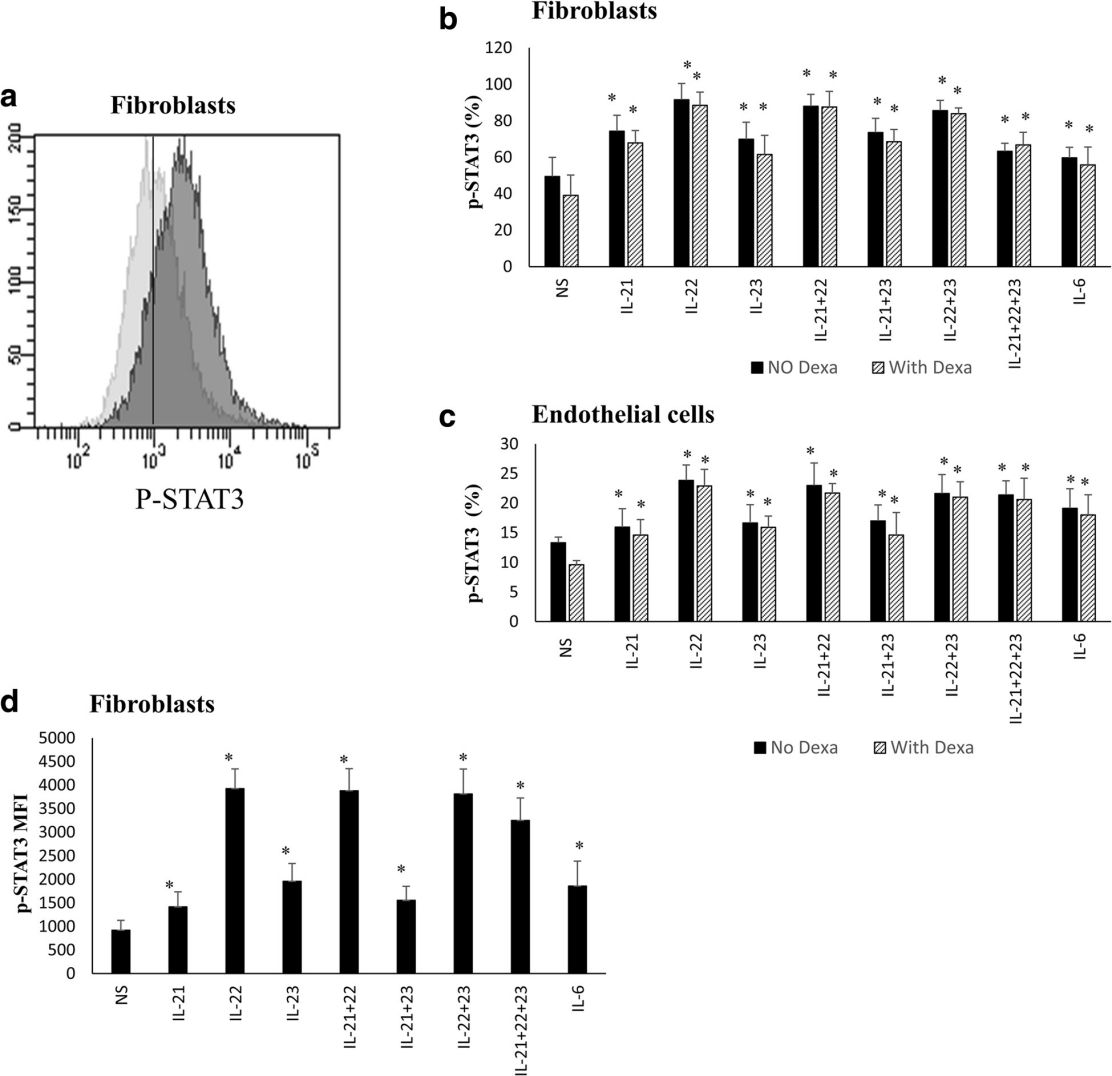
Th-17 regulatory cytokines induce STAT3 phosphorylation in fibroblasts and endothelial cells. Primary human lung fibroblasts and HMVEC-L endothelial cells were treated or not with dexamethasone (5 μM) for 1 h then stimulated with cytokines for 15 min, fixed in 4 % PFA and ice-cold methanol and stained with PE labeled-anti-p-STAT3 antibody and analysed using the BD LSRII flow cytometer. **a** Representative FACS data showing level of STAT3 phosphorylation in fibroblasts following IL-21+22+23 cytokines stimulation. **b, c** Percentage of p-STAT3 following treatment, or not, of fibroblasts (**b**) and endothelial cells (**c**) with cytokines alone or in combinations. **d** Mean Fluorescent Intensity (MFI) of p-STAT3 within fibroblasts following treatment with cytokines. n = 8 for each cell type. Comparison is always between cells treated with cytokines (in the presence or absence of Dexamethasone) and non-treated cells. Data is expressed as means ± SE *p ≤0.05. *NS* non-stimulated

To explore the mechanism behind cytokine-induced cell protection, cultured ASMCs, endothelial cells and fibroblasts treated or not with dexamethasone (5 μM) for 1 h were stimulated with IL-21, 22, and 23 cytokines for 15 min and the frequencies of positive cells for p-STAT3 were determined by FACS analysis. Figure 2a shows representative data for STAT3 phosphorylation of fibroblasts stimulated or not with Th-17 cytokines. STAT3 phosphorylation increased significantly in fibroblasts stimulated with all cytokines alone or in combinations. p-STAT3 phosphorylation levels of cells not treated with dexamethasone were comparable following single or double cytokines stimulations although double cytokine stimulation gave a slightly higher phosphorylation levels (IL-21: 74.4 %, *p* <0.0001, IL-22: 91.5 %, *p* <0.0001; IL-23: 69.8 %, *p* = 0.002; IL-21+22: 87.9 %, *p* <0.0001; IL-21+23: 73.5 %, *p* <0.0001; IL-22+23: 85.6 %, *p* <0.0001; IL-21+22+23: 63.2 %, *p* = 0.007; and IL-6: 59.6 %, *p* = 0.043). Similar results were obtained when cells were previously treated with dexamethasone but with slightly lower levels of STAT3 phosphorylation (IL-21: 67.9 %, *p* <0.0001, IL-22: 88.5 %, *p* <0.0001; IL-23: 61.5 %, *p* = 0.002; IL-21+22: 87.6 %, *p* <0.0001; IL-21+23: 68.5 %, *p* <0.0001; IL-22+23: 83.9 %, *p* <0.0001; IL-21+22+23: 66.8 %, *p* <0.0001; and IL-6: 55.8 %, *p* = 0.011). The combination of the three cytokines induced the lower level of STAT3 phosphorylation. Similarly, stimulating endothelial cells with these cytokines alone or in combinations significantly induced STAT3 phosphorylation (IL-21: 15.9 %, *p* = 0.054, IL-22: 17.8 %, *p* = 0.001; IL-23: 16.6 %, *p* = 0.019; IL-21+22: 17.9 %, *p* = 0.008; IL-21+23: 16.9 %, *p* = 0.005; IL-22+23: 18.6 %, *p* = 0.001; IL-21+22+23: 21.4 %, *p* <0.0001; and IL-6: 19.1 %, *p* = 0.001). Treating cells with dexamethasone did not affect cytokines ability to significantly induce STAT3 phosphorylation (IL-21: 14.6 %, *p* <0.0001, IL-22: 18.9 %, *p* <0.0001; IL-23: 15.9 %, *p* <0.0001; IL-21+22: 16.0 %, *p* <0.0001; IL-21+23: 14.6 %, *p* = 0.005; IL-22+23: 20.0 %, *p* <0.0001; IL-21+22+23: 20.6 %, *p* <0.0001; and IL-6: 18 %, *p* <0.0001) (Fig. 2b). However, none of these cytokines induced significant level of STAT3 phosphorylation in ASMCs cells (data not shown).

**Fig. 3:**
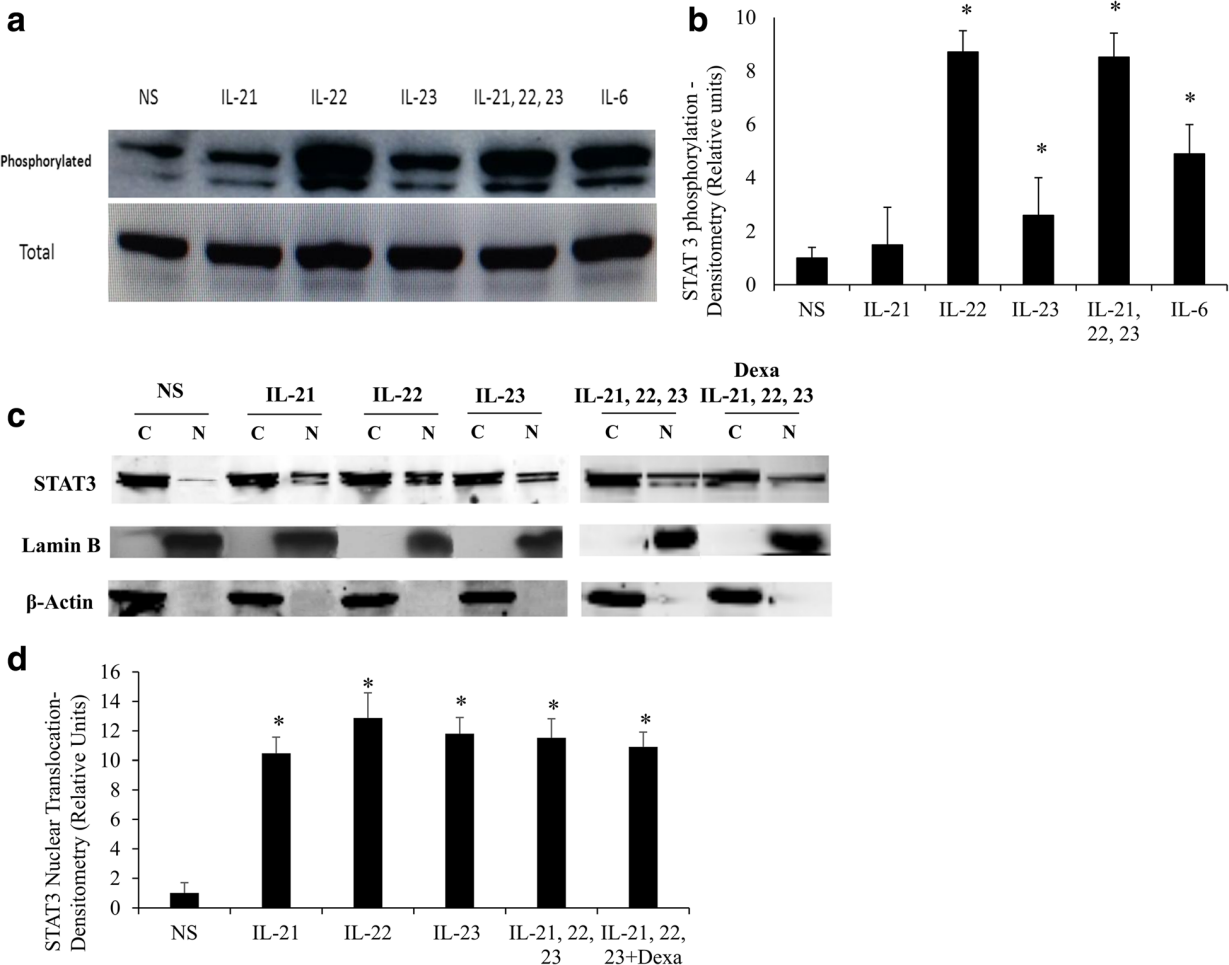
Western analysis confirming phosphorylation and nuclear traslocation of STAT3 protein following stimulation of fibroblasts with IL-21, IL-22 and IL-23 cytokines. Primary human lung fibroblasts were stimulated with IL-21, 22 and 23 cytokines alone or in combination for 15 min, cells lysed, fractionated, and proteins resolved using western blotting. **a** Western blot of cell lysates probed with anti-p-STAT3 and anti-STAT3 (**b**) Densitometry of p-STAT3 immunoreactive bands relative to total STAT3. IL-6 was used as positive control. **c** Western blot of cytoplasmic [C] and nuclear [N] fractions probed with anti-STAT3, anti-Lamin B (nuclear marker), and anti-β-actin antibodies (**d**) Densitometry of nuclear STAT3 immunoreactive bands relative to cytoplasmic STAT3. (*n* = 5). Comparison is always between cells treated with cytokines and non-treated cells. Data is expressed as means ± SE. * *p* ≤0.05. *NS* non-stimulated

Further corroboration for cytokine-induced activation of STAT3 phosphorylation was obtained by immunoblotting performed with whole cell lysates of stimulated fibroblasts using an anti-phospho-STAT3 antibody. All the three tested cytokines along with the combination (IL-21+IL-22+IL-23) induced substantial STAT3 phosphorylation (Fig. 3a). IL-6 was used as a positive control. Densitometry data of the immunoblotting indicated that stimulating cells with IL-22 (8.7 fold, *p* <0.001) and IL-21+IL-22+IL-23 (8.5 fold, *p* <0.001) cytokines caused similar elevations in STAT3 phosphorylation while IL-21 (1.5 fold, *p* = NS) and IL-23 (2.6 fold, *p* = 0.056) did not show much increase compared to non-stimulated cells (Fig. 3b). Nuclear translocation of cytokine induced p-STAT3 was also determined using western analysis performed with cytoplasmic and nuclear cellular fractions. IL-21, 22, and 23 along with triple cytokines stimulations induced STAT3 nuclear translocation in fibroblasts (Fig. 3c). Relative to non-stimulated cells, stimulating fibroblasts with IL-22 induced a higher level of STAT3 nuclear translocation (12.90 folds, *p* <0.001) compared to IL-21 (10.4 folds, *p* <0.001), IL-23 (11.8 folds, *p* <0.001) or triple cytokine treatment (10.8 folds, *p* <0.001), although not to a significant level (*p* = NS). Moreover, pre-treating fibroblasts with dexamethasone did not significantly affect cytokine induced STAT3 translocation. These results confirmed that IL-21, IL-22 and IL-23 cytokines activated STAT3 phosphorylation and nuclear translocation in fibroblasts which may hence counteract the corticosteroid apoptotic effect on these cells.

### STAT3 phosphorylation is required for the anti-apoptotic effect of Th-17 regulatory cytokines on lung structural cells

**Fig. 4:**
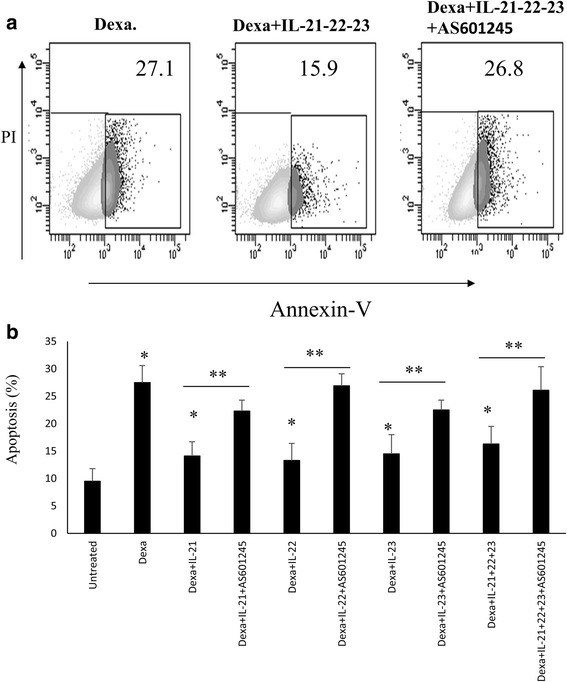
STAT3 phosphorylation is required for IL-21, IL-22, and IL-23 cytokines anti-apoptotic effect on structural cells. Primary human lung fibroblasts were stimulated or not with cytokines in the presence or absence of AS601245 inhibitor and then exposed to dexamethasone 5 μM for 24 h. The percentage of apoptotic cells were quantified by flow cytometry using Annexin V-PE and PI. Apoptotic cells were categorised as being Annexin V+ve but PI-ve. **a** Representative FACS data showing level of apoptosis in dexamethasone treated fibroblasts following IL-21+22+23 cytokines stimulation in the presence or absence of p-STAT3 inhibitor. **b** Level of apoptosis in dexamethasone treated fibroblasts following IL-22, 22, and 23 cytokines stimulation, alone or in combinations, in the presence or absence of p-STAT3 inhibitor (*n* = 7). Data is expressed as means ± SE. Apoptosis of cells treated only with dexamethasone were compared to non-treated cells. Apoptosis of cells treated with Dexamethasone and cytokines were compared to cells treated only with Dexamethasone (* *p* ≤0.05). Apoptosis of cells treated with Dexamethasone and cytokines were compared in the presence or absence of p-STAT3 inhibitor (**p ≤0.05)

To confirm the requirement of Th-17 cytokine-induced STAT3 activation in protecting structural cells from dexamethasone-induced apoptosis, the selective STAT3 inhibitor, AS601245 was used in the presence of cytokines and dexamethasone, as described above. Figure 4a shows representative data of fibroblasts apoptosis following treatment with dexamethasone and Th-17 cytokines in the presence or absence of AS601245. As shown in Fig. 4b, in the absence of STAT3 inhibitor, Th-17 cytokines inhibited dexamethasone induced apoptosis (Dexa: 27.3 %, *p* <0.001; IL-21: 14.1 % *p* = 0.002; IL-22: 13.3 %, *p* = 0.001; IL-23: 14.5, *p* = 0.003; IL-21+22+23:16.3 %, *p* = 0.009). However, when cells were stimulated with Th-17 regulatory cytokines in the presence of the p-STAT3 inhibitor, AS601245, a considerable elevation in apoptosis resumed, with percentage of apoptotic cells (IL-21+AS601245: 22.3 %, *p* = 0.007; IL-22+AS601245: 26.9 %, *p* = 0.001; IL-23+AS601245: 22.5 %, *p* = 0.015; IL-21+22+23+AS601245: 26.1 %, *p* = 0.028). Similar results were obtained for endothelial cells (data not shown). Interestingly, inhibiting IL-22 induced STAT3 phosphorylation restored cells apoptosis to significantly higher levels compared to IL-21 (*p* = 0.055) or IL-23 (*p* = 0.059).

## Discussion

Th-17 regulatory cytokines IL-21, IL-23, IL-6 in addition to IL-22 cytokine were shown to enhance the persistence of various types of cells [47, 55, 56]. The frequency of Th-17 cells and the levels of their regulatory cytokines are upregulated in the airway lung tissue during asthma and COPD relative to the severity of the disease [32, 57–60]. Airway tissue remodelling during asthma is characterized by increased smooth muscle mass, fibrosis, angiogenesis, and mucus production leading to airflow obstruction and increased airway hyper-responsiveness [61]. One of the mechanisms this deformity of airway tissue is achieved with is the enhanced proliferation and persistence of lung structural cells. In this report, we have shown, for the first time, that IL-21, IL-22, IL-23 and IL-6 cytokines significantly inhibit dexamethasone induced apoptosis of cultured airway fibroblasts and endothelial cells. This role of IL-21, IL-22 and IL-23 cytokines in inflamed lung airways may contribute to the persistence of airway remodelling and hence enhance asthma pathogenesis.

To examine the anti-apoptotic effect of IL-21, IL-22, and IL-23 on airway structural cells, their ability to inhibit corticosteroid induced apoptosis was determined. Our data indicated that cytokine treatment was effective in significantly inhibiting induced apoptosis for both fibroblasts and endothelial cells but not ASM cells. Although 50 ng/ml cytokines were used to achieve maximum effect, apoptosis was observed at much lower concentrations (5 ng/ml in most cases). As the levels of Th-17 regulatory cytokines is upregulated especially during severe asthma, this may lead to accumulation of these cytokines to the effective anti-apoptotic levels. On the other hand, while stimulating with double cytokines had a better anti-apoptotic effect than each one alone, combination of all three cytokines had the lowest anti-apoptotic effect especially for fibroblasts. Since the highest anti-apoptotic effect was for IL-22+23 for both cell lines, it seems that when IL-21 is added to IL-22+23, it may trigger a negative feedback mechanism that counteracts their anti-apoptotic activity. IL-21 could stimulate the expression of TNF-α in fibroblasts as it was shown in T cells during Rheumatoid arthritis [62]. Moreover, Juncadella et al. reported recently that the pro-apoptotic effect of TNF-α is synergized in the presence of IL-22 [63]. This may explain the lower anti-apoptotic effect observed when cells are treated with all three cytokines together. Confirming this possibility, however, requires further investigations.

IL-17 and IL-23 cytokines have been shown to promote insensitivity of primary bronchial epithelial cells and peripheral lymphocytes to pro-apoptotic effect of dexamethasone by augmenting GR-β expression [15]. GR-β does not bind known ligands and attenuates the action of GR-α, the hormone binding receptor. Moreover, treating epithelial and lymphoid cells with proinflammatory cytokines TNF-α or IL-1 enhanced the expression and accumulation of GR-β over GR-α receptor via an NF-κB dependent mechanism [64]. The fact that IL-22 and 23 activates NF-κB [65, 66] indicate that they may also favour expression and accumulation of GR-β receptors. This suggest that, in addition to STAT3 activation, other pathways could also contribute to IL-21, 22, and 23 cytokines induced GC insensitivity. Further investigations are needed to unravel these mechanisms which may offer new strategies for therapeutic intervention in GC-insensitive asthma.

STAT3 is a latent cytoplasmic transcription factor that is key regulator of several biological pathways, including differentiation, survival, proliferation, and migration [67]. STAT3 mostly acts as an anti-apoptotic factor, especially in cancerous cells, where it is mostly constitutively active (phosphorylated) [68–70]. In fact, it was suggested that STAT3 can function as an oncogene and is able to transform normal fibroblast cells and cause tumors in nude mice [71]. Binding of IL-21, IL-22, and IL-23 cytokines to their receptors results in the activation of intrinsic receptor tyrosine kinases or receptor-associated tyrosine kinases, such as JAK or SRC. These kinases subsequently phosphorylate the cytoplasmic part of the receptor and provide docking sites for monomeric STATs (STAT1 and STAT3). Once recruited, STAT3 are then phosphorylated on specific tyrosine residues, thus allowing their dimerization and translocation to the nucleus [72]. These STAT3 complexes will then bind to promoters of genes regulating cellular apoptosis such as Bcl-2, Bcl-x and other anti-apoptotic genes and promote their transcription [72].

IL-21, 22, and 23 cytokines induced a significant increase in STAT3 phosphorylation levels in primary fibroblasts and endothelial cells irrespective of their treatment with dexamethasone (Fig. 2). IL-22 and its combination treatments triggered a significantly higher level of STAT3 phosphorylation compared to IL-21 and IL-23 alone (Figs. 2 and 3). The higher level of STAT3 phosphorylation observed for combination treatments especially for IL-21+22 and IL-22+23 (Fig. 2b, c), could also explain, at least in part, the lower level of apoptosis observed following treatment with combination of two cytokines compared to one. Triple cytokines treatment, however, had lower STAT3 phosphorylation levels compared to IL-22 stimulation. Similarly, the level of IL-22 induced STAT3 nuclear translocation was higher compared to that of IL-21 or the triple cytokines stimulations although not to a significant level. This may explain why IL-22 had the higher anti-apoptotic effect compared to other cytokines. Interestingly, inhibiting STAT3 phosphorylation abrogated IL-21, IL-22, and IL-23 anti-apoptotic effect on these structural cells although not to similar levels. The pro-apoptotic effect of p-STAT3 inhibition was highest on cells treated with IL-22 cytokine. Although this data confirm the requirement of STAT3 phosphorylation for IL-21, 22, and 23 anti-apoptotic effect, it does not rule out the involvement of other anti-apoptotic pathways especially for IL-21 and IL-23. This is supported by the fact that contrary to the case of IL-22 where inhibition of STAT3 phosphorylation restored apoptosis to almost Dexamethasone alone levels, IL-21 and 23 restored around 80 % of dexamethasone level. Unraveling the other anti-apoptotic pathways that could be involved requires more investigations.

Several observations have indicated that STAT3 may contribute to the progression of lung fibrosis [72, 73]. Lim and colleagues showed that keloid lesions fibroblasts display constitutive activation of STAT3 signalling, and blocking of this pathway inhibited the profibrotic activity of these cells [75]. Prele et al. demonstrated constitutively active STAT3 of lung fibroblasts isolated from patients with IPF resulted in reduced proliferative capacity, increased expression of anti-apoptotic gene Bcl-xL and Bcl-2, and reduced expression of Thy-1/CD90 and integrin avb3 [76, 77]. The reported herein effect of IL-21, IL-22, and IL-23 on fibroblasts suggest that these cytokines may play a critical role in regulating the survival and probably proliferation of fibroblasts during IPF, via the activation of STAT3, resulting in severe fibrosis. Similarly, they may enhance lung fibrosis in various chronic lung inflammatory diseases especially that the expression of these cytokines in lung tissue during chronic pulmonary inflammation was shown to be elevated. These findings pave the way for more extensive research on the expression and role of these regulatory cytokines in IPF and other chronic inflammatory diseases.

## Conclusions

Lung tissue expression levels of IL-21, IL-22, and IL-23 was previously shown to be upregulated during chronic lung inflammatory diseases. Here we have shown, for the first time, that IL-21, IL-22, and IL-23 cytokines enhance the persistence of lung airway fibroblasts and endothelial cells to corticosteroid induced apoptosis, in a STAT3 dependent manner. This role of Th-17 regulatory cytokines is believed to contribute to the persistence of airway tissue remodelling during chronic inflammatory lung diseases. Knowing that may pave the way for drug interference that may help in preventing or reversing airway lung tissue remodelling especially in severe disease conditions, where such an approach is critically needed.
